# Financial risks of care seeking for malaria by rural households in Jimma Zone, Oromia Region, Southwest Ethiopia: a cross-sectional study

**DOI:** 10.1136/bmjopen-2021-056162

**Published:** 2021-12-30

**Authors:** Lelisa Fekadu Assebe, Dereje Dillu, Gemu Tiru, Kjell Arne Johansson, Sarah Bolongaita, Averi Chakrabarti, Nathaniel Hendrix

**Affiliations:** 1 Global Public Health and Primary Care, University of Bergen, Bergen, Norway; 2 Disease Prevention and Control Directorate, Ministry of Health, Addis Ababa, Ethiopia; 3 Global Health and Population, Harvard T.H.Chan school of Public Health, Harvard University, Cambridge, Massachusetts, USA; 4 Health System Strengthening and Equity Directorate, Ministry of Health, Addis Ababa, Ethiopia

**Keywords:** infectious diseases, health economics, health policy

## Abstract

**Objectives:**

Despite major progress in the prevention and control of malaria in recent years, the disease remains a major cause of morbidity in Ethiopia. Malaria also imposes substantial socioeconomic costs on households. The aim of this study is to estimate the financial risk of seeking malaria service for rural households across socioeconomic statuses in the Jimma Zone, Oromia Region.

**Design:**

A facility-based cross-sectional survey.

**Setting:**

Jimma Zone, Oromia Region, Southwest Ethiopia.

**Participants:**

A total of 221 patients with malaria from 10 public health facilities were interviewed between September 2018 and December 2019.

**Primary and secondary outcome measures:**

The main outcome measures capture the financial risks associated with malaria services, specifically catastrophic and impoverishing health expenditures. Catastrophic health expenditure (CHE) occurs when healthcare costs reach 10% of a household’s monthly income, whereas impoverishment occurs when a household’s monthly income falls below the national poverty level after paying for health service. Descriptive statistics were used to summarise the expenditure patterns associated with malaria services. All costs were gathered in Ethiopian birr and reported in 2019 US$.

**Results:**

The average cost of receiving malaria services was US$4.40 (bootstrap 95% CI: 3.6 to 5.3), with indirect costs accounting for 52% of total costs. Overall, at the 10% threshold, 12% (bootstrap 95% CI: 8.1% to 16.7%) of patients with malaria incurred CHE: 40% (bootstrap 95% CI: 26.7% to 55.6%) of the household in the poorest quintile experienced CHE, but none from the richest quintile did. The proportion of households living in poverty increased by more than 2-3% after spending on malaria-specific health services.

**Conclusion:**

Healthcare seeking for malaria imposes a substantial financial risk on rural households, particularly for the poorest and most vulnerable. Malaria policies and interventions should therefore seek to alleviate both the direct costs and productivity losses associated with the disease, especially among the poor.

**Strengths and limitations of this study**

- The study provided evidence on the financial impact of seeking malaria service in Ethiopia.
- The data were collected using a standard tool and supplemented with consumption data for equity analysis.
- This study looked at both direct and indirect expenses to assess the financial burden of malaria on households, whereas most previous studies only examine the former.
- The presence of multiple malaria episodes with severe consequences may place a greater financial burden on households than a single malaria episode, which we studied here.
- Our findings may not be generalisable to the rest of the country because the study was conducted in a small number of districts.

## Introduction

Malaria is a major public health problem that causes substantial morbidity and mortality worldwide. In 2019, an estimated 229 million malaria cases were reported globally, with the bulk of cases (93%) occurring in Africa.^1^ In Ethiopia, malaria is 1 of the top 10 causes of morbidity,^2^ with an estimated 2.6 million cases and 6000 deaths occurring in 2019.^1^ Nearly 68% of the country’s landmass has altitude and rainfall patterns conducive for malaria transmission and 60% of Ethiopia’s population lives in malaria-prone areas.^3^ Young children and pregnant women are the most affected by the disease.^1^

Ethiopia has made substantial progress in malaria prevention and control in recent years by enhancing access to core malaria interventions in all public health facilities and at the community level. The widespread use of insecticide-treated bed nets and indoor residual spraying and the adoption of artemisinin-based combination therapy (ACT) have been particularly effective at reducing malaria-related morbidity and mortality.^4 5^ As a result, between 2016 and 2019, the annual parasite incidence and mortality of malaria have decreased by 37% and 67%, respectively.^5^ However, the reduction in malaria burden is not uniform subnationally. This is mainly due to diverse ecological risk factors of malaria transmission, differences in utilisation of malaria interventions and development of resistance. These factors pose a challenge to the national goal of malaria elimination.^4^

The economic cost of seeking malaria services can account for a large share of household income and deplete savings for poor households. Furthermore, the seasonal transmission of malaria in countries like Ethiopia overlaps with major farm harvesting and other agricultural activities, resulting in a large loss of productivity, which in turn impacts households’ ability to secure adequate resources for malaria care while also meeting basic consumption needs.^6 7^ In general, malaria costs are low in comparison with other infectious diseases, and less likely to expose households to catastrophic health expenditures (CHEs) and impoverishing expenditures.^6 8–14^ However, the negative impact of malaria on savings, agricultural productivity, investments, and human capital accumulation influences both household welfare and long-term economic growth in the country.^15^ The financial risks resulting from malaria also vary across socioeconomic groups, with the poorest and most vulnerable households bearing the brunt of the burden due to low coverage of any form of prepaid health insurance among this group.^16^ Hence, even relatively inexpensive essential treatments can be burdensome for impoverished households in low-income countries. In addition, the cost of treating malaria in the health system is not negligible and varies depending on the severity of the illness in low-income countries, with Ghana, Tanzania, Kenya, and Mozambique spending US$2.8–123, US$1.75–48, US$2.77–57, and US$4.34–26.56 per uncomplicated case and severe case, respectively.^17 18^

There is, however, limited evidence on the financial risks borne by different socioeconomic groups due to malaria in Ethiopia. Hence, it is important to assess the financial risks (ie, the incidence of catastrophic and impoverishing health expenditures) stemming from seeking malaria services in the country to provide policymakers with insight on the financial risk protection (FRP) afforded by the health system to its population and help to improve health financing system.

## Methods

### Study setting

The study was conducted in Jimma Zone, which is 355 km southwest of Addis Ababa, Ethiopia. The total population of the zone exceeds 3 million people, with the majority (90%) living in rural areas. Jimma Zone is divided into 21 woredas (districts), with a total of 128 public health facilities. It is one of Oromia’s most well-known coffee-growing districts and its plentiful natural resources contribute greatly to the country’s economy.

In most parts of Ethiopia, malaria incidence peaks between September and December, following the main rainy season (June–September), and between March and May, during and after the small rainy season (February–March). Jimma Zone is among the malaria-prone areas, and is located 1780 m above sea level (malaria transmission occurs in Ethiopia at altitudes of up to 2000 m above sea level).^4^

### Study design, sample size and sampling procedure

A health facility-based, cross-sectional survey was carried out in selected health facilities of Jimma Zone, Oromia Region. Data were collected between September 2018 and December 2019.

A minimum sample size of 248 cases (including 10% non-response rate) was estimated with the assumption to detect a mean difference of at least US$3 between each income quintile using an SD of US$5.12^6^ with a 95% CI and a power of 80. Systematic random sampling was employed to select 10 public health facilities from the zone. The number of patients recruited from each facility was proportionate to the annual malaria caseload in each study site. Patients with malaria who attended selected health facilities throughout the study period were interviewed consecutively at the time of health facility exit until the desired sample size for each study site was met.

### Data collection and procedure

A questionnaire adapted from the ACT consortium was used to capture household malaria expenditure.^19^ The questionnaire collected data on sociodemographic variables, household income, consumption, and direct and indirect treatment costs. The value of household consumption items, such as food, non-food and consumer durables, was solicited with varying reference period according to the frequency of purchase. A follow-up phone call to all study participants was later conducted to collect data on the malaria-specific expenses that occurred within a month of the health facility visit. Data collected from study participants were augmented with clinical data (eg, test type for malaria diagnosis, malaria species, medication type, quantity, duration, etc) by reviewing health facility registers.

The questionnaire was translated into the Oromiffa language and back translated to English to ensure consistency. Data collectors received training on the study’s purpose and survey instrument.

### Cost analysis

A cost-of-illness analysis was conducted from the patient’s perspective to estimate the direct and indirect costs incurred with seeking malaria services. Direct costs were defined as the expenses incurred when seeking healthcare services and included two components: direct medical and direct non-medical costs. Direct medical cost covered the cost for consultation or medical registration, testing and diagnosis, medication and other medical . Direct non-medical costs included expenses related to transportation, food and lodging while seeking malaria services.

Indirect costs referred to the productivity losses that occurred in a household during a malaria episode. Patients were asked to estimate working days lost while seeking malaria services, including travel time to and from health facilities. This information was used to compute indirect costs using the human capital approach.^19^ Specifically, the time spent while seeking malaria services (in minutes or hours) was converted to days, with a day defined as 8 working hours (ie, 8 hours=1 day). Time lost (in days) was then multiplied by a daily wage rate, which was derived from monthly consumption, assuming 22 working days per month. Furthermore, all reported food, non-food and durable consumption figures were aggregated to construct household monthly consumption and used as a proxy for household monthly income (ie, hereafter referred to as household income). The household income was then used to group all households into five equal quintiles. Malaria-related costs were analysed separately for individuals in these different quintiles. All costs were gathered in Ethiopian birr (ETB) and converted to US dollars (US$) using the exchange rate during the study period (ie, US$1=31.5 ETB).^20^

### Measuring catastrophic health expenditure and impoverishment

#### Catastrophic health expenditure

Cases of CHE occur when malaria-related expenditures exceeded 10% of household monthly income. Two scenarios of CHE were assessed: the first includes only direct costs (scenario 1), while the second includes both direct and indirect costs (scenario 2). The proportion of households with CHE ( H ) is mathematically estimated as follows:

(1) $$H=\frac{1}{n}\sum_{i=1}^{n}E_{i}$$

Where

$E_{i}=$ indicator function of CHE;

$$E_{i}=\left\{1\;if\;\frac{T_{i}}{y_{i}}\geq z;\;0\;if\;\frac{T_{i}}{y_{i}}§lt;z\right\}$$

$T_{i}=$ household payment for malaria care

$y_{i}=$ total monthly household income

n=

z=

Furthermore, two metrics, mean overshoot (O) and mean positive overshoot (MPO), were used to evaluate the extent or intensity of CHE (ie, the extent to which healthcare costs surpass the income threshold).

The mean overshoot reflects how far malaria-related expenses exceeded the threshold ( z ) across all households, which was calculated as follows:

(2) $$O=\frac{1}{n}\sum_{i=1}^{n}O_{i}$$

Where the overshoot $O_{i}$ was estimated as follows: $O_{i}=(E_{i}(\frac{T_{i}}{y_{i}})-z)$ .

The MPO measured the payment above the threshold ( z ) averaged across all households with CHE. Thus, MPO was equal to:

(3) $$MPO=\frac{O}{H}$$

### Impoverishment

To investigate the impact of malaria healthcare payments on poverty, we first identified the relevant poverty line: the Ethiopian national poverty line of US$28.26 per month.^21^ Next, we estimated how the households of individuals in the study sample fared on four poverty measures before and after malaria-specific healthcare payments. Specifically, we analysed: (1) the poverty headcount ratio or the proportion of households living below the poverty line; (2) the total poverty gap or the aggregate distance from the poverty line across all households; (3) the normalised poverty gap, a measure computed by dividing the average poverty gap by the poverty line, which makes easier to compare the poverty gaps computed across different poverty lines (areas) and (4) the mean positive poverty gap, or the average consumption shortfall of the poor.

The pre-payment poverty headcount ( $H^{pre}$ ) was the total number of poor households among the total sample, calculated as:

(4) $$H^{pre}=\frac{1}{n}\sum_{i=1}^{n}P_{i}^{pre}$$

Where

$$Pi^{pre}=\left\{1\left(poor\right)ify_{i}<z^{pre};0ify_{i}\geq z^{pre}\right\}$$

$z^{pre}=$ the pre-payment poverty line

The pre-payment poverty gap (depth of poverty) was calculated using the formula:

(5) $$G^{pre}=\frac{1}{n}\sum_{i=1}^{n}{g_{i}}^{pre}$$

Where

The normalised pre-payment poverty gap expressed the total poverty gap as multiples of the poverty line, and was defined as:

(6) $$NG^{pre}=\frac{G^{pre}}{z^{pre}}$$

The mean positive pre-payment poverty gap was given by the following:

(7) $$MNG^{pre}=\frac{G^{pre}}{H^{pre}}$$

Post-payment poverty measures (corresponding to the time after malaria service costs had been incurred) were constructed analogously. The difference between the pre-payment and post-payment poverty values of each measure provides an estimate of the poverty impact of malaria healthcare payments on households (equations 4–7).

### Data analysis

Data were analysed using R statistical software, V.4.^22^ Descriptive statistics were used to summarise continuous data, whereas frequency counts and percentages were presented for categorical data. The outcome measures of interest were malaria-specific patient costs, catastrophic and impoverishing health expenditures. Total costs (ie, direct and indirect costs combined) were divided by the number of households that incurred malaria expenditures to arrive at the average patient cost per malaria treatment. A bootstrap with 10 000 iterations was performed to estimate the 95% CIs for the costs. We used concentration curves and indices to examine whether costs related to malaria impose disparities on households in different socioeconomic groups. A pen’s parade plot was used to compare the income distribution before and after malaria care costs among households ranked from poorest to richest. We tested the statistical significance of all differences in outcomes between income quintiles with the Kruskal-Wallis test. Furthermore, a sensitivity analysis was performed, to ensure the generalisability of our findings to the national context, by grouping study participants into national income quintiles (derived from Ethiopia’s Gross domestic product (GDP) per capita of US$855.8 and its Gini index of 0.332) using gamma distribution rather than sample-specific income quintiles (online supplemental file, tables S1 and S2).^23 24^

10.1136/bmjopen-2021-056162.supp2 Supplementary data

### Patient and public involvement

Patients or the public were not involved in the design, conduct, reporting or dissemination plans of this research.

## Results

A total of 221 patients with malaria or their caregivers were interviewed (table 1). The mean age of study participants was 23 years (SD 14.74). Most participants (91%) lived in rural areas and about two-thirds (64%) were male. Households in the study were comprised of 1–18 members, with the typical household having five members. The mean household monthly income was US$79 (SD 53.18). The average duration of malaria illness before seeking care was 3 days (SD 2), and 80% of the study participants had no history of malaria illness. More than three-fourths (83%) reported having access to malaria health services within 5 km of their homes. Nearly half (51%) of the study participants were infected with *Plasmodium falciparum* species of malaria parasite.

**Table 1:** Sociodemographic, economic and clinical characteristics of study participants (N=221), 2019

Variables	Category	Frequencies (%)
Gender	Male	141 (63.8)
	Female	80 (36.2)
Family size	≤4	85 (38.5)
	>4	136 (61.5)
Place of residence	Rural	202 (91.4)
	Urban	19 (8.6)
Marital status	Single	115 (52.0)
	Married/living together	100 (45.2)
	Divorced and separated	4 (1.9)
	Widowed	2 (1.0)
Highest level of education	No education	85 (38.5)
	Elementary (grade 1–8)	112 (50.7)
	Secondary and higher	24 (10.9)
History of malaria infection in the past years	Yes	45 (20.4)
	No	176 (79.6)
Type of malaria	*Plasmodium falciparum*	112 (50.7)
	*P. vivax*	109 (49.3)
Mean age in years (SD)		23 (14.7)
Average distance to health facility in kilometres (SD)		4 (5.1)
Mean duration of illness in days (SD)		3 (1.9)
Mean monthly household income in US$ (SD)	Q1	22 (8.4)
	Q2	48 (6.5)
	Q3	72 (7.7)
	Q4	97 (6.9)
	Q5	157 (57.3)

Q1=poorest income quintile; Q5=richest income quintile.

### Costs borne by patients in obtaining treatment for malaria

The average total cost of seeking care for a malaria episode was US$4.4 (bootstrap 95% CI: 3.6 to 5.3) (table 2). Of the total costs, direct medical costs typically accounted for 23% (US$1.0), non-medical costs accounted for 25% (US$1.1) and productivity loss accounted for 52% (US$2.3). Drug and transportation costs (particularly for households without access to malaria services within 5 km), at US$0.6 per malaria case, account for the largest share of both direct medical and direct non-medical costs, respectively. Total patient costs amounted to an average of 6% of monthly household income. We found evidence of varying malaria costs across socioeconomic status: the cost for households in the richest quintile was US$6.3, while for those in the poorest quintile was US$2.5 (p=0.015). These differences were entirely driven by indirect costs. We also found that patient costs varied significantly across malaria species, with infections with *P. falciparum* costing more than infections with *P. vivax* (online supplemental table S3).

**Table 2:** Patient costs by cost category in aggregate and across socioeconomic groups, 2019

	Cost categories					
	Direct		Indirect		Total	
Income quintile	Mean (95% CI)	Median (95% CI)	Mean (95% CI)	Median (95% CI)	Mean (95% CI)	Median (95% CI)
Q1	2.1 (1.6 to 2.5)	1.7 (1.3 to 2.3)	0.4 (0.3 to 0.5)	0.3 (0.2 to 0.4)	2.5 (2.0 to 3.0)	2.1 (1.7 to 2.6)
Q2	2.2 (1.4 to 3.1)	1.1 (0.7 to 2.3)	2.4 (1.2 to 4.0)	0.7 (0.4 to 0.9)	4.6 (3.0 to 6.5)	2.4 (1.7 to 3.1)
Q3	2.0 (1.4 to 2.8)	1.2 (0.9 to 1.8)	1.9 (1.0 to 3.3)	0.8 (0.6 to 0.9)	3.9 (2.6 to 5.6)	2.3 (1.6 to 2.9)
Q4	2.4 (1.6 to 3.4)	1.3 (1.0 to 1.8)	2.3 (1.2 to 4.0)	1.2 (0.9 to 1.5)	4.7 (3.0 to 7.1)	2.4 (2.2 to 3.0)
Q5	1.8 (1.3 to 2.4)	1.3 (0.9 to 1.6)	4.5 (2.4 to 7.5)	2.1 (1.6 to 2.9)	6.3 (4.0 to 9.3)	3.7 (3.0 to 4.8)
Total	2.1 (1.8 to 2.4)	1.3 (1.1 to 1.6)	2.3 (1.7 to 3.1)	0.8 (0.7 to 1.0)	4.4 (3.6 to 5.3)	2.5 (2.2 to 2.9)
Concentration index		−0.03		0.27		0.13
P value*		0.808		<0.001		0.015

*Kruskal-Wallis test.

#### Financial burden imposed by malaria and inequalities across income quintiles

In table 3, we show that the proportion of households experiencing CHE as a result of malaria was 12% under scenario 1 (ie, payments for malaria-related medical services while receiving care) and 19% under scenario 2 (ie, both direct and indirect costs for malaria services). Among those experiencing CHE, the mean positive overshoot under the two scenarios was 18% and 19%. This represents the proportion of additional payments for malaria care that surpasses 10% of total consumption in households with CHE. As expected, the poorest income quintiles experienced higher incidence and intensity of CHE (p<0.001).

**Table 3:** Incidence and intensity of catastrophic health expenditure (CHE) among patients with malaria across income quintiles, 2019

	Income quintile					Total	P value*
	Q1	Q2	Q3	Q4	Q5		
Scenario 1: direct costs							
CHE incidence	0.40	0.11	0.02	0.07	0.0	0.12	<0.001
Overshoot	0.092	0.010	0.003	0.002	0.0	0.020	
Mean positive overshoot	0.231	0.097	0.138	0.034	0.0	0.181	
Scenario 2: direct and indirect costs							
CHE incidence	0.44	0.27	0.11	0.09	0.05	0.19	<0.001
Overshoot	0.104	0.045	0.015	0.012	0.007	0.040	
Mean positive overshoot	0.234	0.163	0.129	0.131	0.156	0.190	

*Kruskal-Wallis test.

Table 4 documents the impoverishing effects of malaria expenditures. About 2% of study participants’ households were pushed below the national poverty line by direct malaria costs (scenario 1). When total costs were considered (ie, direct and indirect costs, scenario 2), this proportion rose to 3%. The normalised poverty gap increased from 5% of the poverty line to 6% and 7%, respectively, when direct and total malaria costs were compared of household income. However, the normalised mean positive poverty gap increased only slightly from 8.9 to 9.8 when considering only direct costs and to 10.1 when considering total costs. This indicates that while a small number of households are being driven into poverty by malaria-related health expenditures, those who are currently poor are made poorer by the disease.

**Table 4:** Incidence and intensity of impoverishing health expenditure among patients with malaria across income quintiles, 2019

	Income quintile					Total
	Q1	Q2	Q3	Q4	Q5	
Scenario 1: direct costs						
Poverty headcount (%)						
Pre	75.6	0	0	0	0	15.4
Post	84.4	2.3	0	0	0	17.6
Net	8.8	2.3	0	0	0	2.2
Poverty gap (US$)						
Pre	6.8	0	0	0	0	1.4
Post	8.3	0.2	0	0	0	1.7
Net	1.5	0.2	0	0	0	0.3
Normalised poverty gap (%)						
Pre	23.9	0	0	0	0	4.9
Post	29.5	0.5	0	0	0	6.1
Net	5.6	0.5	0	0	0	1.2
Mean positive poverty gap (US$)						
Pre	8.9	0	0	0	0	8.9
Post	9.9	6.5	0	0	0	9.8
Net	1.0	6.5	0	0	0	0.9
Scenario 2: direct and indirect costs (total cost)						
Poverty headcount (%)						
Pre	75.6	0	0	0	0	15.4
Post	84.4	6.8	0	0	0	18.6
Net	8.8	6.8	0	0	0	3.2
Poverty gap (US$)						
Pre	6.8	0	0	0	0	1.4
Post	8.7	0.5	0	0	0	1.9
Net	1.9	0.5	0	0	0	0.5
Normalised poverty gap (%)						
Pre	23.9	0	0	0	0	4.9
Post	30.8	1.6	0	0	0	6.6
Net	6.9	1.6	0	0	0	1.7
Mean positive poverty gap (US$)						
Pre	8.9	0	0	0	0	8.9
Post	10.3	6.8	0	0	0	10.1
Net	1.4	6.8	0	0	0	1.2

Poverty line (monthly)=US$28.26.

**Figure 1:**
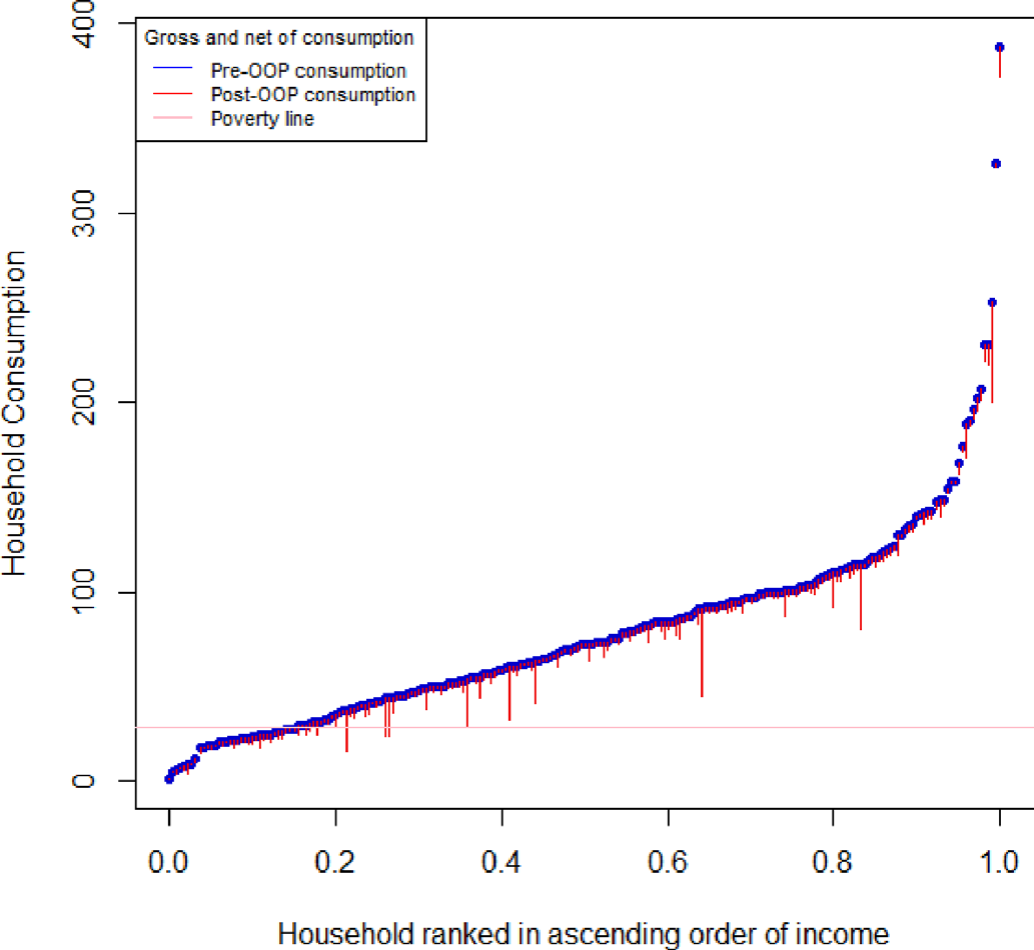
Pen’s parade of household consumption (US$) gross in terms of multiple of the poverty line and net of healthcare payments. OOP, out-of-pocket.

The spikes or ‘paint drips’ in the pen’s parade plot in figure 1 reveal the extent to which the subtraction of total malaria-specific costs (scenario 2) reduces household income. The total cost of malaria resulted in a slight reduction in household income but was not substantial enough to greatly increase poverty levels.

**Figure 2:**
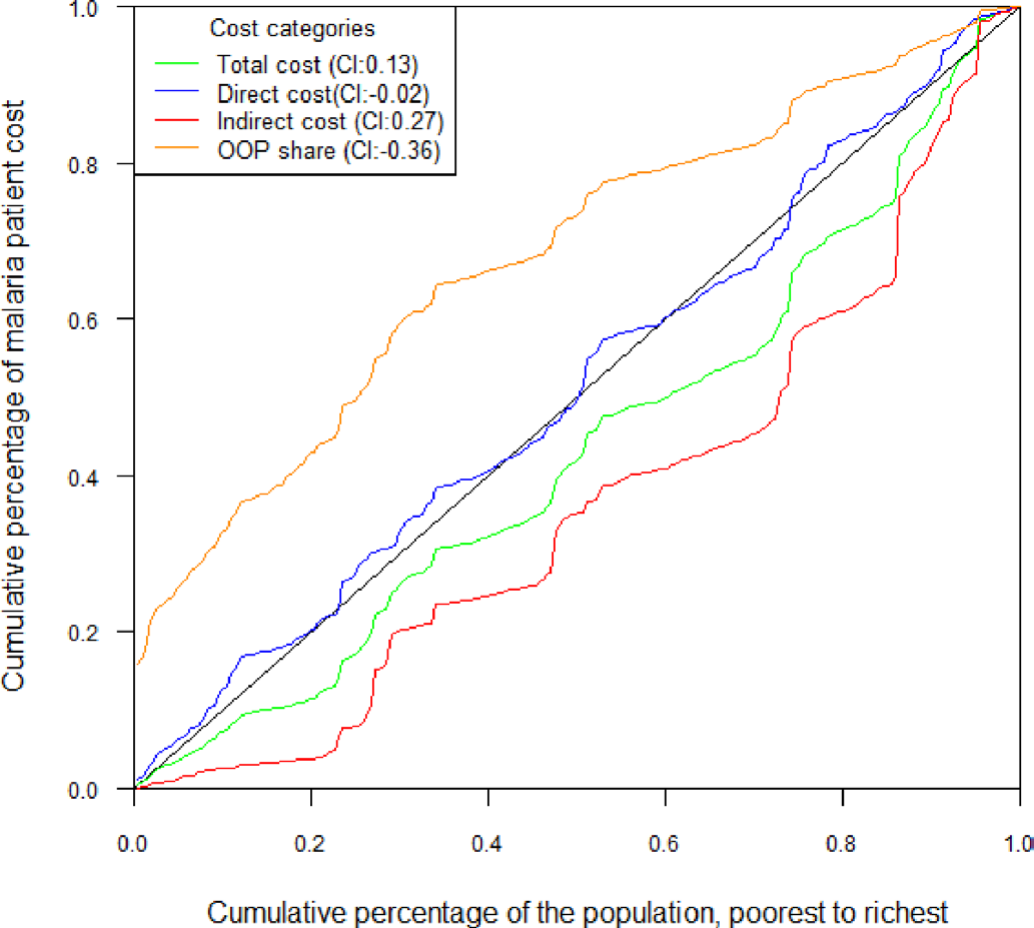
Concentration curve plotting the cumulative percentage of patient cost or share of OOP against household income ranked in ascending order. Figures in parentheses represent concentration indices (I). OOP, out-of-pocket.

Finally, we present the relationship between the cost of obtaining malaria healthcare and household income in the form of a concentration curve and index in figure 2. The concentration curve and index show that total and indirect costs were concentrated among the rich (concentration index, 0.13 and 0.27), while the income share of out-of-pocket costs was concentrated among the poor (concentration index, −0.36). The curve for direct costs virtually overlaps with the line of equality, which indicates that direct costs did not vary significantly by household income.

## Discussion

Universal health coverage (UHC) aims to improve access to quality services while providing financial risk protection by reducing or eliminating direct payments for health services.^25^ However, patient costs continue to be a major obstacle to the achievement of UHC. In this analysis, we seek to provide economic estimates of the cost imposed by malaria in order to determine the FRP needs associated with the disease and for effective guidance of resource allocation.

The average cost of malaria treatment in this study was $4.40, despite the fact that all public health facilities in Ethiopia provide free malaria prevention and treatment. Direct non-medical and productivity loss-related expenses account for the majority of malaria expenditures, rather than direct medical costs which are largely covered by the government. These expenses have considerable economic implications for households. For example, rural household members might have to withdraw from harvesting and other agricultural activities during an illness, thus leading to low productivity.^8^ A majority of the Ethiopia’s population are at risk of malaria^3^ and need to pay to receive care for the disease. This potentially jeopardises a household’s capacity to afford treatment when faced with multiple morbidities. Concerningly, healthcare payments for malaria are more likely to result in CHE and impoverishment among the poorest households. At the broadest level, malaria imposes significant economic externalities: it dampens school attendance and performance, interferes with the accumulation of human and physical capital, and likely reduces tourism and/or foreign investment.^15^

The estimated total cost of malaria services in our study is similar to what has been reported previously in other parts of Ethiopia (US$5.06 per malaria case) and Ghana (US$4.91 per malaria case).^6 7^ However, a study conducted in the Chewaka District of Western Ethiopia found a higher mean annual cost of malaria disease of US$16.^26^ The variation could be due to the method employed to examine costs (particularly indirect costs) and the differences in study settings. More than half of the total malaria costs identified in our study were attributed to productivity-related expenses, which is consistent with other findings.^6 26 27^ This calls for optimising universal coverage of malaria prevention, diagnostics and treatment services, as well as improving social protection schemes (eg, income support, illness insurance and transportation vouchers) to move towards malaria-free goals.^28^

In this study, the incidence of CHE resulting from malaria expenses stood below 20%, as has been shown elsewhere.^29^ Our result also demonstrates that malaria treatment pushed 2%–3% of households below the national poverty line, which is troublesome given that more than a quarter of the Ethiopia’s population lives in poverty.^21^ In general, our findings demonstrate the FRP value of malaria control programmes as compared with other diseases programmes.^9 10^ The Ethiopian government’s commitment, combined with support from development partners, allows for enhanced access to malaria services and helps to reduce the prevalence of malaria and protect households from financial risk.^30^ As a result of these efforts, for instance, most of the study participants were able to access malaria services within a 5-kilometre radius, reducing the cost of travelling and delayed care seeking. It is worth pointing out here that a previous study in Malawi showed that people who live within 5 km of a health facility have a 40% lower cost per malaria episode than those who live further away.^31^

Our results also documented that the financial burden imposed by malaria differs substantially across the income spectrum. Specifically, we found support for previous findings that indicate that malaria costs are regressive, with the poor spending a higher proportion of their income than their wealthier counterparts.^16^ Malaria services are prohibitively expensive for poor households that are least likely to be equipped to protect themselves against the disease and its recurrence. These households have to meet the cost of malaria care out of their scarce resources, and this may further impoverish them^32^ and their vulnerability to poverty is further influenced by several factors (such as the impact of foreign direct investment, tourism, labour productivity, etc).^33^ Understanding the various mechanisms through which malaria causes poverty is essential to understand so that future malaria control and elimination efforts can guard against such eventualities.

There are several limitations to our study. First, a household may experience more than one malaria episode per year or severe malaria episodes. Unfortunately, we are unable to speak to the potential cost of repeat or severe malaria infections. These contingencies would impose a larger financial strain on households than the single malaria episodes we studied here. Second, our results do not represent those households experiencing malaria infections, but are unable to seek care (ie, the very poor). Third, even if our sensitivity analysis showed fewer differences when national income is used, our findings may not be generalisable to the rest of Ethiopia because the study was conducted in a small number of districts. Fourth, because the study was cross-sectional, causal inferences on CHE and poverty outcomes could not be determined.

Despite the limitation, analyses of disease-specific FRP, such as the one we conducted here, have several advantages. First, estimates of population-level-aggregated financial risks differ substantially from those identified by disease-specific studies.^29^ Second, tropical diseases tend to have unique characteristics (such as those related to disease course and treatment duration and efficacy) that necessitate specific interventions for control or elimination. Third, we examined patient expenditures in a comprehensive and systematic way, considering both direct and indirect expenses, as well as the implications of these costs (catastrophic and impoverishing expenditures) across socioeconomic status.

## Conclusion

Malaria imposes a substantial financial risk on rural households, particularly on poor and vulnerable households. This study showed that the financial burden incurred in seeking malaria treatment can lead to catastrophic health expenditures and impoverishment for some households. This calls for a comprehensive social protection scheme, one that covers both direct and indirect expenses and thereby protects households from the financial risks associated with the multitude of costs associated with malaria disease.

10.1136/bmjopen-2021-056162.supp1 Supplementary data

## Data Availability

Data are available upon reasonable request. The datasets used and/or analysed during the current study are available from the corresponding author upon reasonable request.
