# Supplementary material for: Financial risks of care seeking for malaria by rural households in Jimma Zone, Oromia Region, Southwest Ethiopia: a cross-sectional study
Source: BMJ Open. 2021 Dec 30;11(12):e056162. doi: 10.1136/bmjopen-2021-056162 (PMC8719194; doi:10.1136/bmjopen-2021-056162)
Supplement: Supplementary data [file bmjopen-2021-056162supp002.pdf]

Supplementary file

Table S1. Patient costs by cost category (aggregate and across socio-economic groups) using national quintiles, 2019.

	Cost categories					
	Direct		Indirect		Total	
Income quintile	Mean (95% CI)	Median (95% CI)	Mean (95% CI)	Median (95% CI)	Mean (95% CI)	Median (95% CI)
Q1	1.9 (1.4-2.5)	1.2 (1.0-1.6)	1.7 (1.1-2.5)	0.9 (0.7-1.4)	3.6 (2.8-4.7)	3.0 (2.1-3.3)
Q2	2.0 (1.3-2.9)	1.1 (0.8-1.7)	2.7 (1.4-4.4)	0.9 (0.6-1.6)	4.7 (3.1-6.6)	2.4 (1.9-3.3)
Q3	2.1 (1.5-2.9)	1.4 (1.2-1.9)	2.0 (1.0-3.6)	0.9 (0.4-1.5)	4.1 (2.6-6.5)	2.4 (2.0-3.1)
Q4	2.6 (1.9-3.6)	1.7 (1.1-2.8)	2.9 (1.1-5.7)	0.7 (0.5-1.1)	5.6 (3.4-8.6)	2.8 (2.0-4.1)
Q5	1.7 (1.2-2.3)	1.0 (0.7-1.7)	2.1 (1.0-4.0)	0.8 (0.6-1.1)	3.8 (2.4-5.7)	2.3 (1.8-2.9)
Total	2.1 (1.8-2.4)	1.3 (1.1-1.6)	2.3 (1.7-3.1)	0.8 (0.7-1.0)	4.4 (3.6-5.3)	2.5 (2.2-2.9)
Concentration index		-0.001		0.03		0.016
p-value*		0.144		0.676		0.749

* Kruskal-Wallis test, CI: Confidence Interval

Table S2. Incidence and intensity of catastrophic health expenditure among malaria patients across income quintiles, 2019.

	Income quintile						
	Q1	Q2	Q3	Q4	Q5	Total	p-value*
Scenario 1: Direct costs							
CHE incidence	0.38	0.09	0.05	0.04	0.0	0.11	<0.001
Overshoot	0.054	0.012	0.004	0.001	0.0	0.014	
Mean positive overshoot	0.145	0.140	0.091	0.040	0.0	0.131	
Scenario 2: Direct and indirect costs							
CHE incidence	0.73	0.29	0.14	0.13	0.07	0.28	<0.001
Overshoot	0.136	0.053	0.022	0.019	0.003	0.05	
Mean positive overshoot	0.186	0.180	0.162	0.146	0.054	0.17	

* Kruskal-Wallis test

Table S3: Total patient costs by malaria species, 2019.

Cost	Plasmodium falciparum		Plasmodium vivax	
	Mean (95% CI)	Median (95% CI)	Mean (95% CI)	Median (95% CI)
Total cost (US$)	5.6 (4.2-7.2)	3.0 (2.6-3.7)	3.1 (2.6-3.8)	2.1 (1.9-2.4)
Kruskal-Wallis chi-squared = 6.9294, df = 1, p-value = 0.008479				
